# Wind velocity field estimation from aircraft derived data using Gaussian process regression

**DOI:** 10.1371/journal.pone.0276185

**Published:** 2022-10-31

**Authors:** Marius Marinescu, Alberto Olivares, Ernesto Staffetti, Junzi Sun

**Affiliations:** 1 School of Telecommunication Engineering, Rey Juan Carlos University, Fuenlabrada, Madrid, Spain; 2 Faculty of Aerospace Engineering, Delft University of Technology, Delft, The Netherlands; Fuzhou University, CHINA

## Abstract

Wind velocity field knowledge is crucial for the future air traffic management paradigm and is key in many applications, such as aircraft performance studies. This paper addresses the problem of spatio-temporal windc velocity field estimation. The north and east wind components within a given air space are estimated as a function of time. Both wind velocity field reconstruction in space for a past or present time instant and short-term prediction are performed. Wind data are obtained indirectly from the states of the aircraft broadcast by the Mode-S and ADS-B aircraft surveillance systems. The Gaussian process regression method, which is a flexible and universal estimator, is employed to solve both problems. Under general conditions, the method is statistically consistent, meaning that the method converges to the ground truth when increasingly more data are available, which is especially interesting, since aircraft data availability is expected to grow in the future through the deployment of the European System-Wide Information Management. Besides estimation, the Gaussian process regression method provides the probability distribution of any particular estimate, allowing confidence intervals to be computed. Moreover, the spatial modelling is performed using raw data without relying on grids and estimation can be performed at any spatio-temporal location. Furthermore, since the training phase of the method described in this paper is fast, requiring less than 5 minutes on a standard desktop computer, it can be used online to continuously track the state of the wind velocity field, thus allowing for data assimilation. In the case study presented in this paper, the Gaussian process regression method is tested on different days with different wind intensities. The available data set is split into several training and testing data sets, which are used to check the consistency of the results of wind velocity field reconstruction and prediction. Finally, the Gaussian process regression method is validated using the European Centre for Medium-Range Weather Forecasts ERA5 meteorological reanalysis data. The obtained results show that Gaussian process regression can be used to reliably estimate the wind velocity field from aircraft derived data.

## 1 Introduction

The Air Traffic Management (ATM) system has a very complex structure consisting of multiple and heterogeneous components, in which uncertainty is ubiquitous and inevitable [1]. More specifically, four main sources of uncertainties can be distinguished: inaccurate data, decisions made by pilots and air traffic controllers, equipment malfunctioning, and weather conditions.

Weather is one of the most important sources of uncertainty in the ATM system. According to a recent article of the Federal Aviation Administration (FAA) [2], adverse weather conditions is the main cause of air traffic delay in the USA, being the cause of about 70% of delays, whereas in Europe, almost half of the delays are due to adverse weather conditions [3]. The cost for airlines of an hour delay ranges from about 1,400 to 4,500 dollars. Moreover, extreme weather events may compromise safety in air traffic operations and cause disruption to air traffic flow. In this sense, the American Meteorological Society recommends using probabilistic forecasts to consider the intrinsic uncertainty in meteorological predictions [4].

Meteorological information will be even more decisive in the future ATM paradigm, where Four-Dimensional (4D) aircraft trajectories are to be predicted with high precision [5]. In the last decades, air traffic has doubled every 15 years worldwide and the tendency is to increase. There are several ongoing projects, such as the Single European Sky ATM Research (SESAR) in Europe and the Next Generation Air Transportation System (NextGen) in the USA, the aim of which is satisfying the future ATM system requirements through the incorporation of new emerging technologies. Improving capacity, efficiency, safety, and reducing costs and environmental impact are their main objectives, in which the central element is the concept of Trajectory-Based Operations (TBO).

The TBO concept considers 4D aircraft trajectories, which consist in precise descriptions of aircraft paths in space and time. Time delays are considered as deviations of the trajectory in the same way as for horizontal or vertical deviations. TBO improve the strategic planning of aircraft traffic to reduce imbalances between demand and capacity. Additionally, TBO enable the ATM system to know and, when appropriate, modify the aircraft planned trajectories before or during the flight, with the aim of optimising them and improving air space capacity and efficiency of the ATM system.

Trajectory predictability, which is the correspondence between planned and actual trajectory, is a key aspect for TBO, since this concept of operations requires high precision in aircraft trajectory tracking. Multiple random factors, such as uncertainty on weather conditions, mainly wind and storms, diminish precision, which results in deviations from the reference trajectory. Thus, to improve trajectory predictability, precise wind information is necessary [6, 7].

Nowadays, aircraft trajectory planning mostly relies on winds forecasts from Numerical Weather Prediction (NWP) models [8]. In general, NWP meteorological forecasts have a coarse spatial resolution and a low update rate, which is usually every 6 h. The observations used in NWP models are mainly gathered from ground stations and weather balloons, which are launched from specific locations no more than four times per day. Although existing technologies such as the Meteorological Routine Air Report (MRAR) allow aircraft meteorological data to be incorporated into NWP models, currently, MRAR direct wind observations are only returned by a small fraction of aircraft. A consequence of their coarse spatial resolution is that NWP meteorological forecasts suffer from over-smoothness, i.e., local variations are overlooked, which makes the use of NWP inadequate for TBO, as pointed out in [5, 9].

A possible solution to increase the spatial and temporal resolution of wind forecasts is using aircraft derived data [10], the availability of which is expected to grow in the future through the deployment of the European System-Wide Information Management (SWIM), which consists in services based on a unified infrastructure to exchange flight information among all stakeholders [11].

In this article, Mode-S and ADS-B aircraft derived data are used for spatio-temporal wind velocity field estimation. Mode-S is an interrogation protocol and ADS-B is a surveillance technology that broadcast aircraft flight states, such as position, Mach number, ground speed, airspeed, and roll and heading angles. A detailed description of these technologies can be found in [12]. Wind velocity is obtained indirectly from the state of the aircraft, specifically using the vector relation between airspeed, ground speed, and wind velocity.

To deal with non-conventional meteorological observations, such as aircraft derived data, atmospheric data assimilation techniques can be employed. Atmospheric data assimilation is concerned with devising techniques for combining different information sources to estimate the state of the atmosphere, and is a well-established area of research [13]. However, most of the atmospheric data assimilation techniques are focused on assimilating non-conventional meteorological data into NWP models [14, 15]. On the contrary, the data assimilation technique used in this article do not involve NWP. It is based on statistical principles, allowing the large amount of recorded aircraft data to be exploited for wind velocity field estimation. According to the statistics from Flightradar24, in 2019, more than 10,000 aircraft were flying across the globe at any time, resulting in an average of approximately 190,000 flights per day.

This article concerns about spatio-temporal wind velocity field estimation using aircraft derived wind observations. The north and east wind components are estimated as a function of time within a given air space, such as a Terminal Manoeuvring Area (TMA), which is the air space around a major airport. Specifically, a cuboidal region of base size 500 × 500 km centred at the Adolfo Suárez Madrid-Barajas (LEMD) airport with altitude ranging from 0.6 km to 14 km, has been considered.

The wind is modelled as a random field with space and time as inputs and wind velocity components as outputs. The data are considered to be partial observations of the full random field. For this random field, two estimations can be carried out: estimation in space, which can be performed over a past or present time instant (reconstruction), and estimation in time (prediction or forecast). In this paper, the wind velocity field is continuously reconstructed over an hour time period, and the provided forecast is a short time horizon forecast, usually referred to as nowcast in meteorology.

In [14], it has been shown that assimilation of Mode-S wind and temperature observations has a positive impact on a regional NWP model. Similarly, Aircraft Meteorological DAta Relay (AMDAR) and Aircraft Communication Addressing and Reporting System (ACARS) data have been used in [15] with clear benefits on the performance of short and medium range NWP forecasts.

In [7], using B-splines, aircraft wind derived data have been used to reconstruct the wind profile, which has been employed to update optimal aircraft descent trajectories in real-time. In [16], wind uncertainty has been represented by means of a statistical model, in which the correlation of four distinct sources of uncertainty has been included and a filter has been used to describe the evolution of wind uncertainty with time, with the aim of measuring the impact of wind prediction uncertainty on aircraft trajectory prediction. The capability of Kriging, a geostatistical technique, to generate short-term weather predictions along aircraft trajectories has been tested in [17]. An innovative model, combining particle filtering and Lagrangian transportation modelling, has been used in [12] for partial weather field reconstruction.

The contribution of this paper is to provide a method capable of efficiently assimilating aircraft derived wind observations with the aim of accurately reconstructing the wind velocity field and performing short-term wind velocity field predictions. Unlike previous approaches such as [7, 18, 19], in which only wind speed is estimated, and only in a one-dimensional profile, in this paper both north and east wind velocity components are estimated in an entire region using an adaptation of the Gaussian Process Regression (GPR) method. The version of the GPR method implemented in this paper is iterative and fast, enabling data assimilation. Moreover, the classical GPR method only estimates scalar outputs, whereas in this paper, the method is adapted to estimate two related quantities: the components of the wind velocity. To the best of the authors’ knowledge, who employed the GPR method for wind speed estimation in [19], the GPR method has not been used for this purpose yet.

To check the robustness of GPR method, it is tested in several scenarios. Wind velocity observations corresponding to two different days with different wind behaviour are selected for testing. The first day has weaker wind with larger directional dispersion whereas the second day has stronger wind with lower direction dispersion.

For each day, the data set is split in training and test sets in two different manners, namely randomly selecting a set of individual observations and randomly selecting a set of flights and using all the observations collected during these flights.

In addition, the wind velocity field reconstruction obtained through the GPR method is compared with the European Centre for Medium-Range Weather Forecasts (ECMWF) ERA5 meteorological reanalysis data. The results obtained are consistent with reanalysis data, proving the capability of the GPR method to estimate wind velocity in regions of the air space with no or a reduced number of observations.

The method presented in this paper for wind velocity field estimation based on aircraft derived data contains significant improvement and generalisation of previous approaches. The training phase of the method proposed in this paper is faster than the training phase of the method employed in [17], which is not very suitable for nowcasting. Moreover, the method proposed in this paper provides estimates over an entire air space together with a measure of the uncertainty of the estimates, whereas in the method described in [12] the estimates are only available on grid points with data or in the vicinity of these points.

The paper is organized as follows. In Section 2, an exploratory analysis of the data set is performed. In Section 3, the wind velocity field estimation method is explained. In Section 4, the model set up is described and the reconstruction and prediction of the wind velocity field are discussed. In Section 5, the GPR method is validated comparing the obtained results with a reanalysis data set. Finally, the significance of the obtained results are discussed in Section 6.

## 2 Data set preparation and exploratory analysis

### 2.1 Data set preparation

In this paper, data from the All-Purpose Structured EUROCONTROL Surveillance Information Exchange (ASTERIX) library [20], has been used, which have been provided by ENAIRE, the Spanish air navigation service provider. The ASTERIX data set contains a great amount of information, as described in the technical documents of EUROCONTROL, the European Organisation for the Safety of Air Navigation.

In particular, the ADS-B [21] and Mode-S [22] information has been extracted from the ASTERIX data base, and, when necessary, the python library pyModeS [23] has been employed to decode the messages. ADS-B messages carry information about aircraft position and ground speed, whereas Mode-S messages carry information related to aircraft state as is detailed in [24]. Both messages are broadcast with a high refresh rate [25] and can be openly received by researchers around the world with inexpensive receivers.

**Fig 1:**
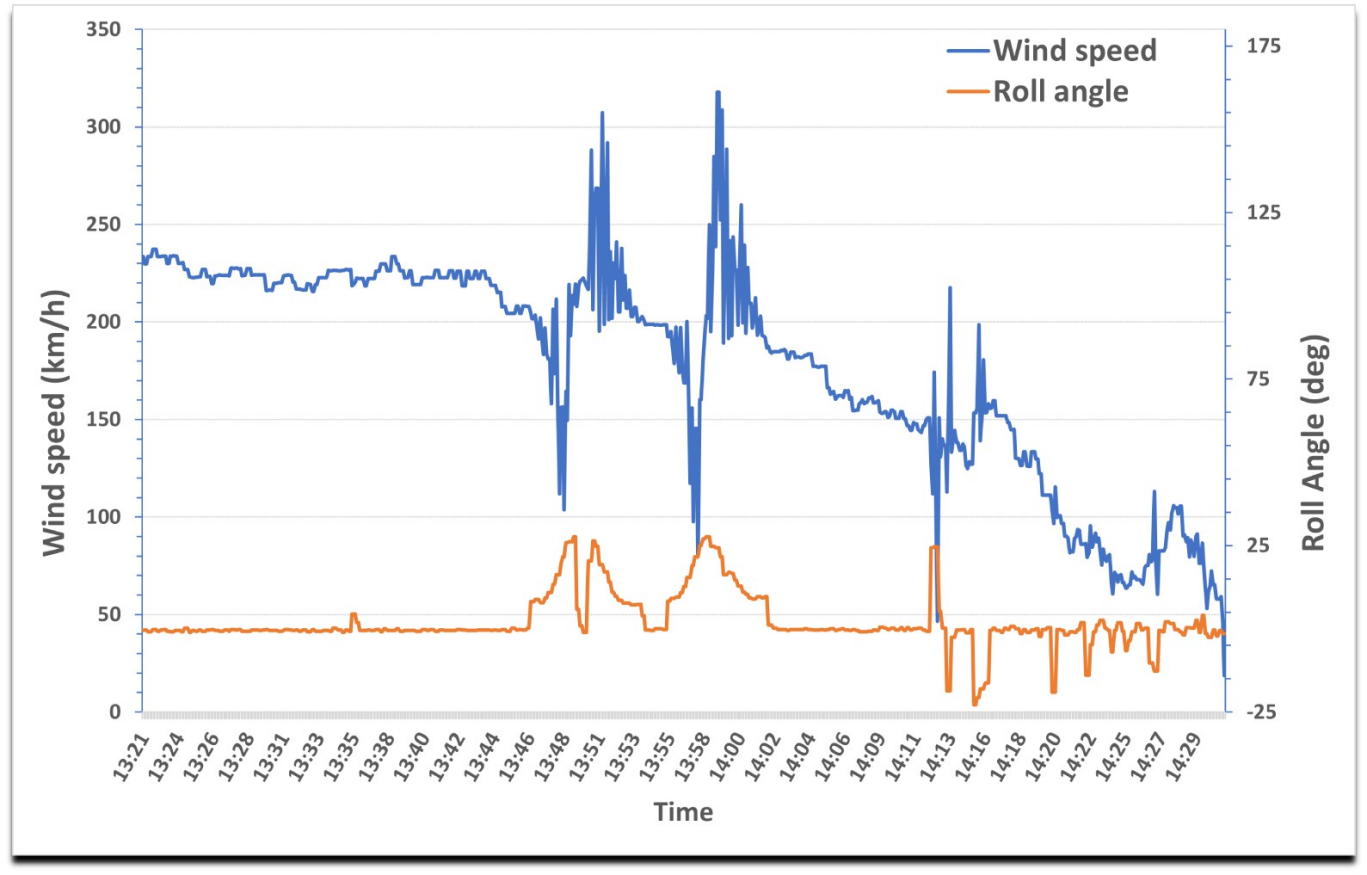
Roll angle and wind speed versus time of an aircraft.

Once the decoded messages were obtained, the ADS-B and Mode-S data were merged into a single data set. For each ADS-B message, the closest Mode-S message within a 5-second range has been sought. If no Mode-S observation was found, the ADS-B row was disregarded. To avoid redundant data, consecutive ADS-B messages having the same Mode-S observation have been eliminated. Corrupted messages have also been removed from the data set, along with noisy data coming from aircraft during non-steady flight phases, outliers, and ground or close to ground observations. More specifically, the following observations have been removed:

- Noisy data: Wind observations collected during turning manoeuvres, in which the roll angle of the aircraft is nonzero, are less reliable [10]. In Fig 1, aircraft’s roll angle and wind speed are represented versus time. It can be seen that the noise in wind speed increases when the aircraft reach high roll angles. Therefore, data collected with roll angles greater than 5° have been removed.
- Outliers: Data have been grouped based on the altitude at which they have been collected. Specifically, a vertical extent of 1 km has been considered for this grouping. Within each group, the usual interquartile range method for outlier detection and removal has been applied. They correspond to outliers commonly seen in a boxplot.
- Ground or close to ground observations: Ground or low altitude data may produce inaccurate wind measurements. Therefore, data collected at altitudes below 640 m above sea level have been removed from the data set, since the data considered in this article have been derived from aircraft flying in the TMA of the LEMD airport, which is located at the altitude of 610 m above the sea level.

### 2.2 Exploratory data analysis

In this section, the main characteristics of the aircraft derived wind observations are described, including measures of centrality and dispersion. Two sets of data collected from 08:00 to 14:00 UTC on two different days are considered:

- A data set with lower wind speed and higher variance in wind direction, collected on February 23, 2020. This data set is referred to as Day 1.
- A data set with higher wind speed and lower variance in wind direction, collected on December 21, 2019. This data set is referred to as Day 2.

The motivation behind the choice of these two days for the numerical experiments is to test the proposed wind velocity field estimation method in two different wind scenarios regarding wind intensity and direction. More specifically, the Spanish State Meteorological Agency [26] historical weather data base has been explored, searching for the days of 2019 and 2020 with highest average wind speed and greatest variance in wind direction around the LEMD airport. Once the two days satisfying these requirements have been identified, the corresponding Mode-S and ADS-B aircraft derived data have been selected from the ASTERIX data base.

Notice that the observations of the two data sets have been collected from 08:00 to 14:00 UTC, which is the time period with the highest air traffic density around the LEMD airport. In particular, the number of observations in the the Day 1 data set is 223.456, whereas this number rises to 232.357 in the Day 2 data set. This amount of observations is large enough for the proposed wind velocity field estimation method based on GPR. Training and building a proper GPR model from few data would be a more challenging problem requiring devising a particular technique, which is out of the scope of this paper.

**Fig 2:**
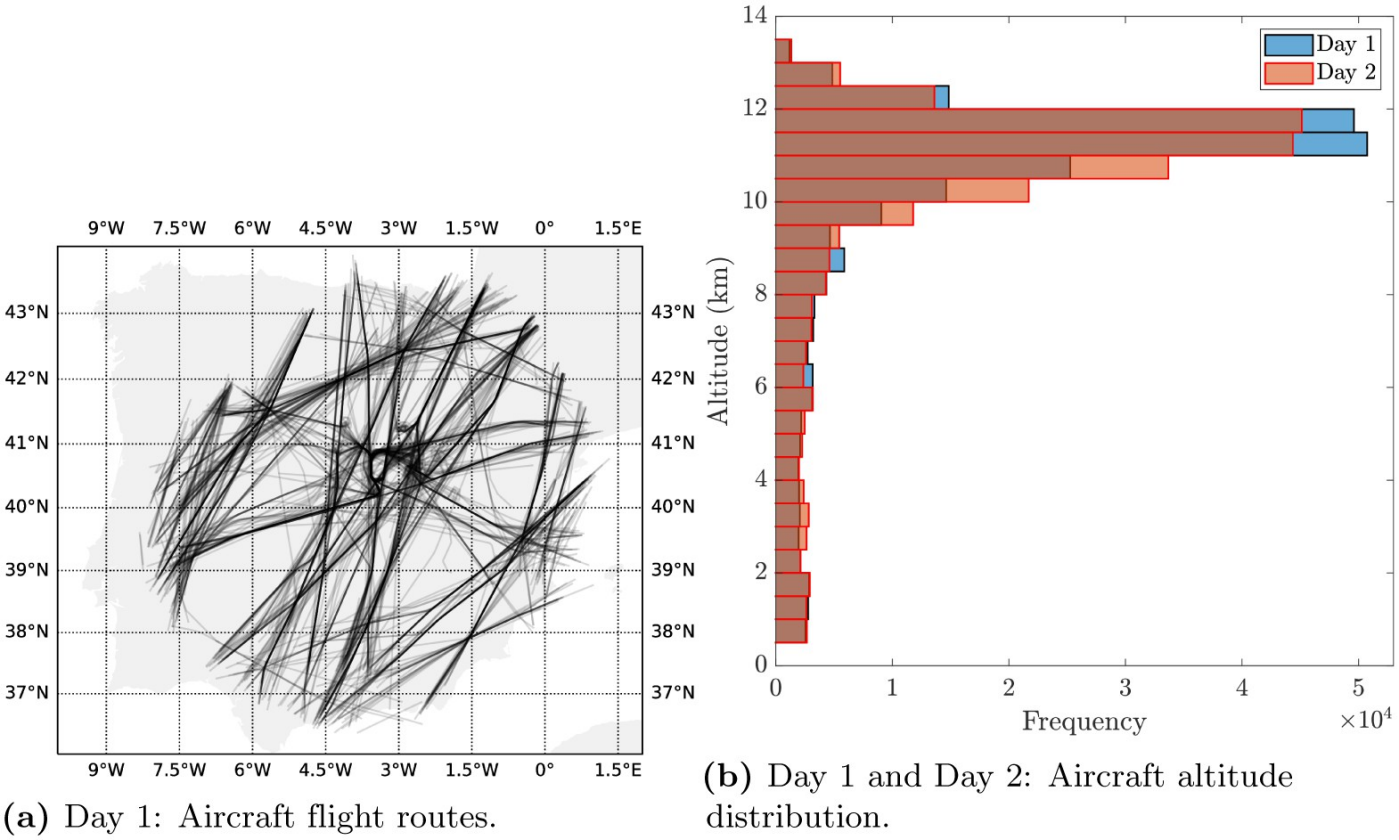
Spatial distribution of the data. (**a**) Day 1: Aircraft flight routes. (**b**) Day 1 and Day 2: Aircraft altitude distribution.

The spatial distribution of the data is represented in Fig 2. More specifically, Fig 2a shows the coverage region over the Iberian peninsula, along with the flight routes, of the Day 1 data set and Fig 2b shows the distribution of the altitudes of both the Day 1 and Day 2 data sets. It can be seen that most of the aircraft are flying at cruise level. In general, the data are highly non-uniformly distributed in the air space, making the estimation more challenging.

**Fig 3:**
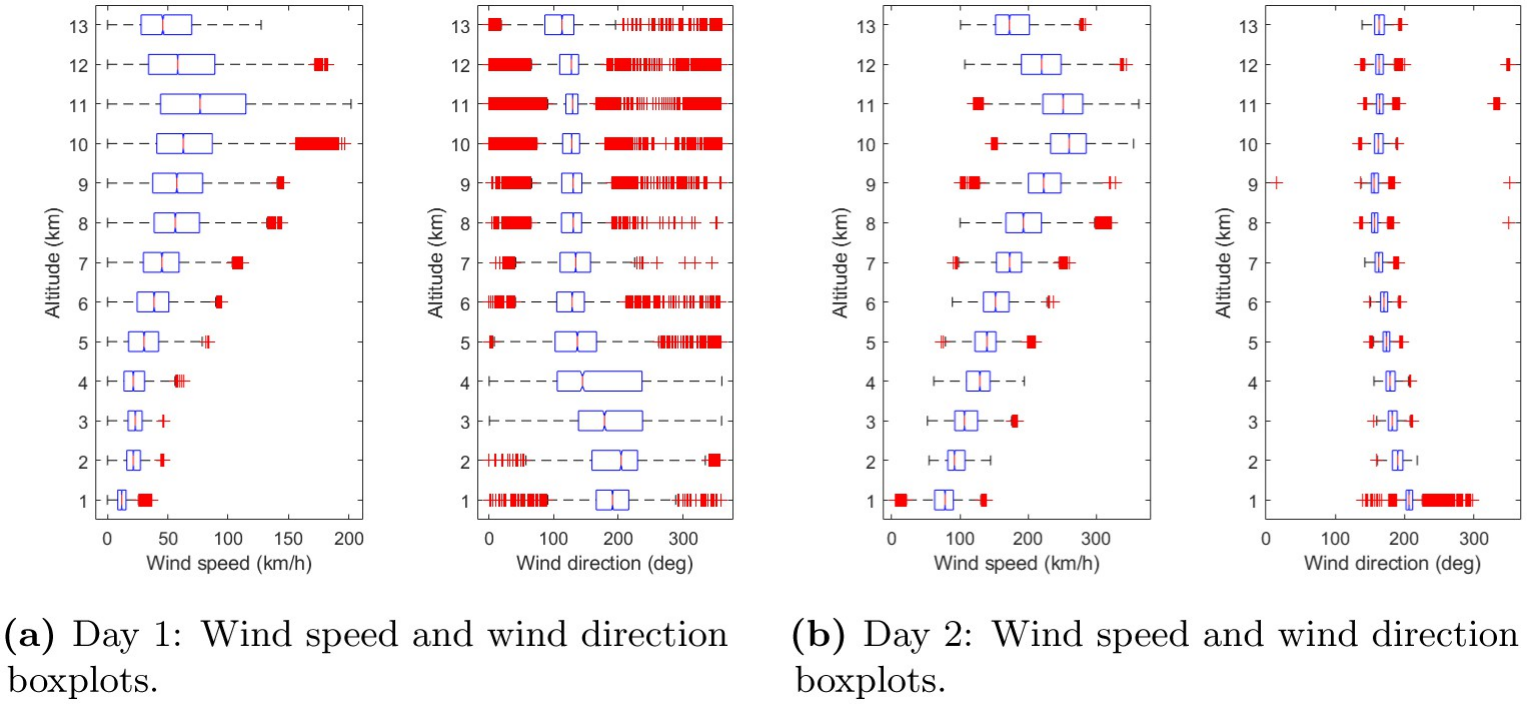
Wind speed and direction distribution grouped by altitudes. Angles in the Day 1 data set are turned 180 deg for an easier interpretation of the boxplots. (**a**) Day 1: Wind speed and wind direction boxplots. (**b**) Day 2: Wind speed and wind direction boxplots.

The boxplots of the wind speed and direction, grouped by altitudes, for the Day 1 and Day 2 data sets are shown in Fig 3. Notice that the outliers shown this figure are new outliers which appeared after the removal of original outliers as explained in Section 2.1.

There are significant differences in the wind features of these two data sets. In particular, in the Day 1 data set, low wind speeds are present at each altitude, with a median value of 16 m/s (56 km/h) and a maximum value of 56 m/s (202 km/h). The dispersion of the wind direction is high, ranging from 0 to 360 deg and only above 6 km of altitude the dispersion of the wind direction decreases. Conversely, in the Day 2 data set, the median wind speed is 63 m/s (226 km/h) with a maximum of 102 m/s (365 km/h) and there is no wind intensity close to zero, except for the first kilometre of altitude. The dispersion of the wind directions is lower in comparison with the Day 1 data set, and the ranges of the boxplots are about 50 degrees. In both data sets, wind intensity grows until 10-11 km of altitude and decreases afterwards.

**Fig 4:**
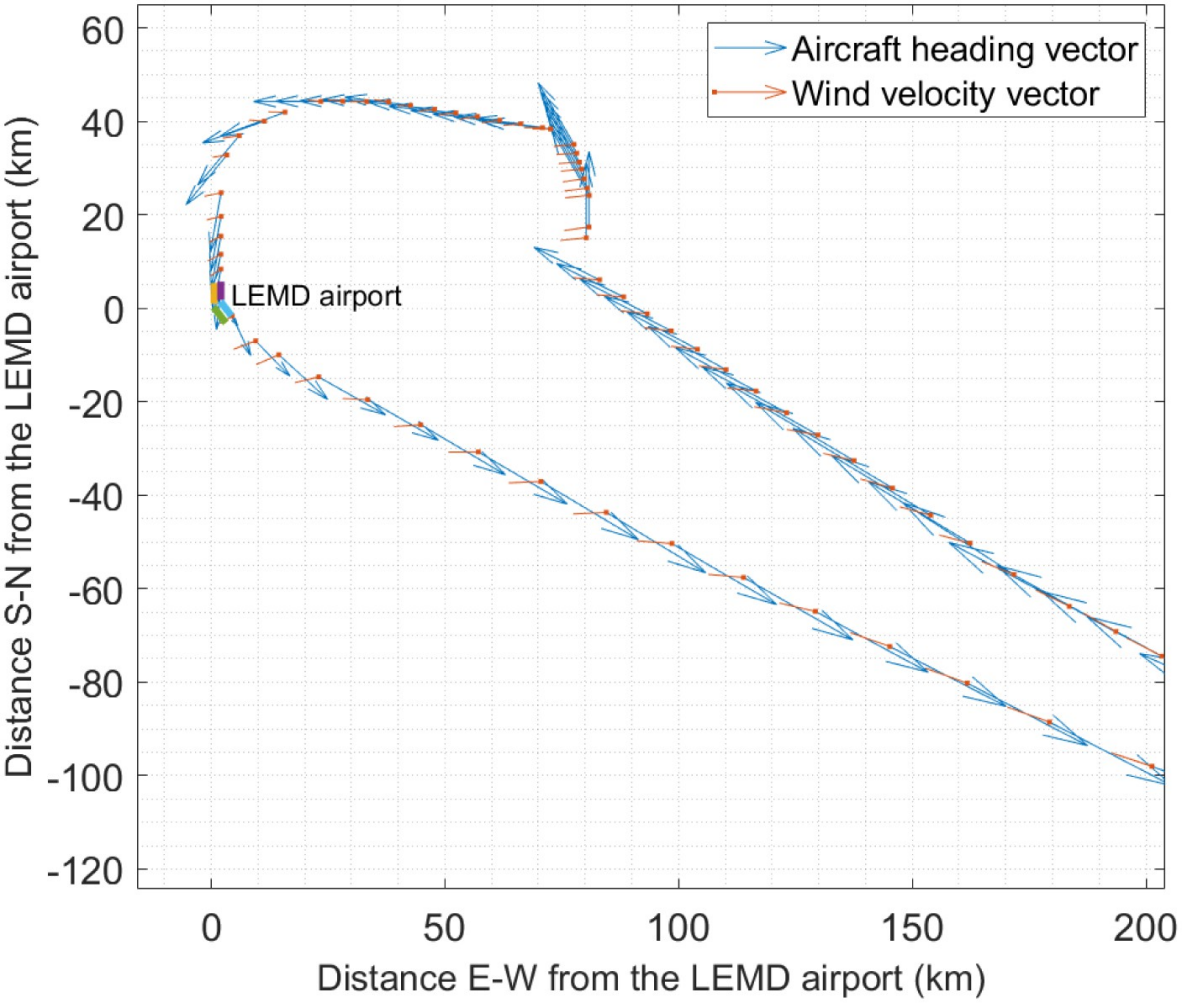
Day 2: Aircraft heading vectors and wind velocity vectors at different positions along an arrival and a departure routes of an aircraft.

The positions of an aircraft landing at the LEMD airport are shown in Fig 4 together with the aircraft heading vectors and wind velocity vectors. The aircraft comes from south-east, turns counterclockwise and arrives at the airport. After 2 h and 15 min the aircraft departs in the south-easterly direction. It can be observed that the aircraft heading vector is not always aligned with the aircraft’s direction of motion (track vector) due to the drift caused by the wind. For this aircraft, the median difference between the directions the heading and track vectors is 3.33 deg. However, in the worst case, the difference between the directions of the heading and track vectors reaches 20.039 deg. In general, for all the observations, the median difference between the directions of the heading and track vectors is 3.165 deg for the Day 1 data set, whereas this value rises to 13.184 deg for the Day 2 data set.

The wind statistics for each day are summarised in Table 1. The mean and variance for angles are computed using circular statistics [27]. It can be observed that the mean wind speed in the Day 2 data set is more than three times higher than the mean wind speed in the Day 1 data set, but the dispersion in wind direction is much lower, around 10 times less.

**Table 1:** Summary of wind statistics.

	Wind speed (m/s)		Wind direction (deg)	
	Day 1	Day 2	Day 1	Day 2
Min.	0	0.013	0.01	163.79
Max.	56.04	100.75	359.99	351.55
Mean	17.80	60.56	307.16	166.66
Dispersion	11.30	16.67	19.40 (%)	2.11 (%)

**Fig 5:**
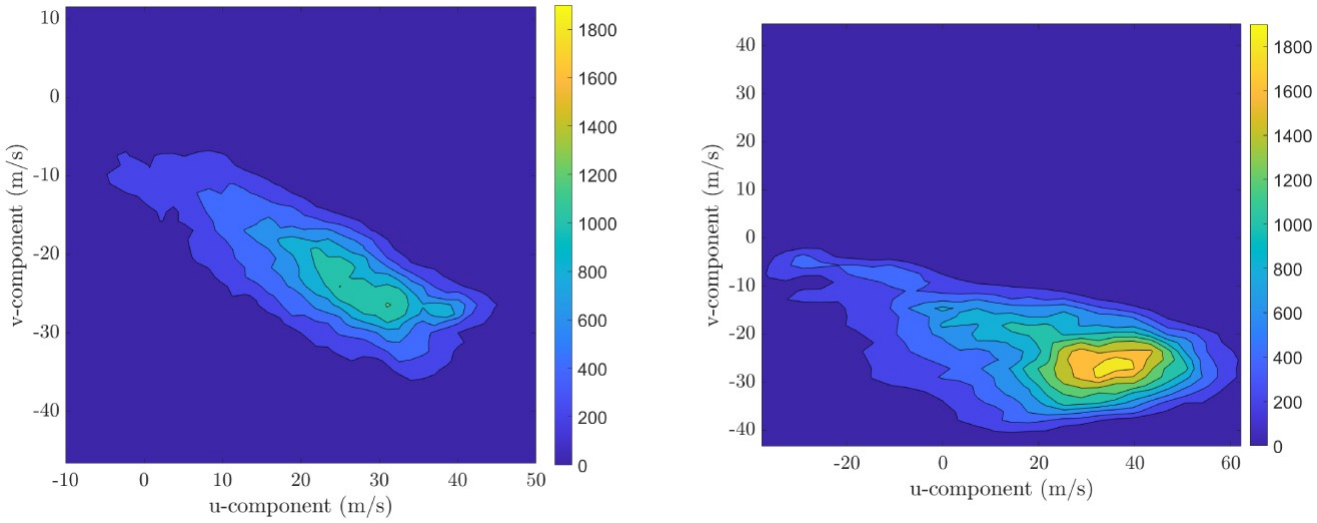
Contour plots of the joint empirical PDF of the *u* and *v* components of the wind velocity for Day 1 (left) and Day 2 (right) data sets. The empirical correlations for Day 1 and Day 2 data sets are $\hat{\rho }=-0.7\pm 0.0021$ and $\hat{\rho }=-0.47\pm 0.0032$, respectively.

Lastly, the statistical relation between the components of the wind velocity is studied. Let *u* and *v* denote the north and east components of the wind velocity. Fig 5 shows the contour plots of the joint empirical PDF of the *u* and *v* components of the wind velocity for both data sets. The empirical correlations for Day 1 and Day 2 data sets are $\hat{\rho }=-0.7\pm 0.0021$ and $\hat{\rho }=-0.47\pm 0.0032$, respectively, indicating that there is statistical dependence between the *u* and *v* components of the wind velocity. The statistical dependence is more predictable linearly for the Day 1 data set than for the Day 2 data set. In general, for a given wind data set, there is statistical dependence, although this statistical dependence usually varies with the spatial location, altitude, and time instant.

The covariance of a single component of the wind velocity at two different spatio-temporal locations is referred to as auto-covariance. In this article, the auto-covariance structure is modelled through the so-called kernel function, which allows a variety of spatio-temporal covariance structures to be modelled. The modelling of the cross-covariance between the *u* and *v* components of the wind velocity is a more delicate task, which will be discussed in sections 3.1 and 3.2.

## 3 Gaussian process regression

GPR is an effective tool that can be seen as a general regression model, which is used in a wide variety of disciplines, including machine learning [28]. Gaussian stochastic processes are capable of modelling a wide range of patterns such as linearity, periodicity, symmetry, continuity, differentiability, non-differentiability, or smoothness. They can be fully determined by the mean function and the covariance function, which is also known as the kernel of the process. The mean function models the deterministic trend of the process, whereas the covariance function models its stochastic properties.

**Fig 6:**
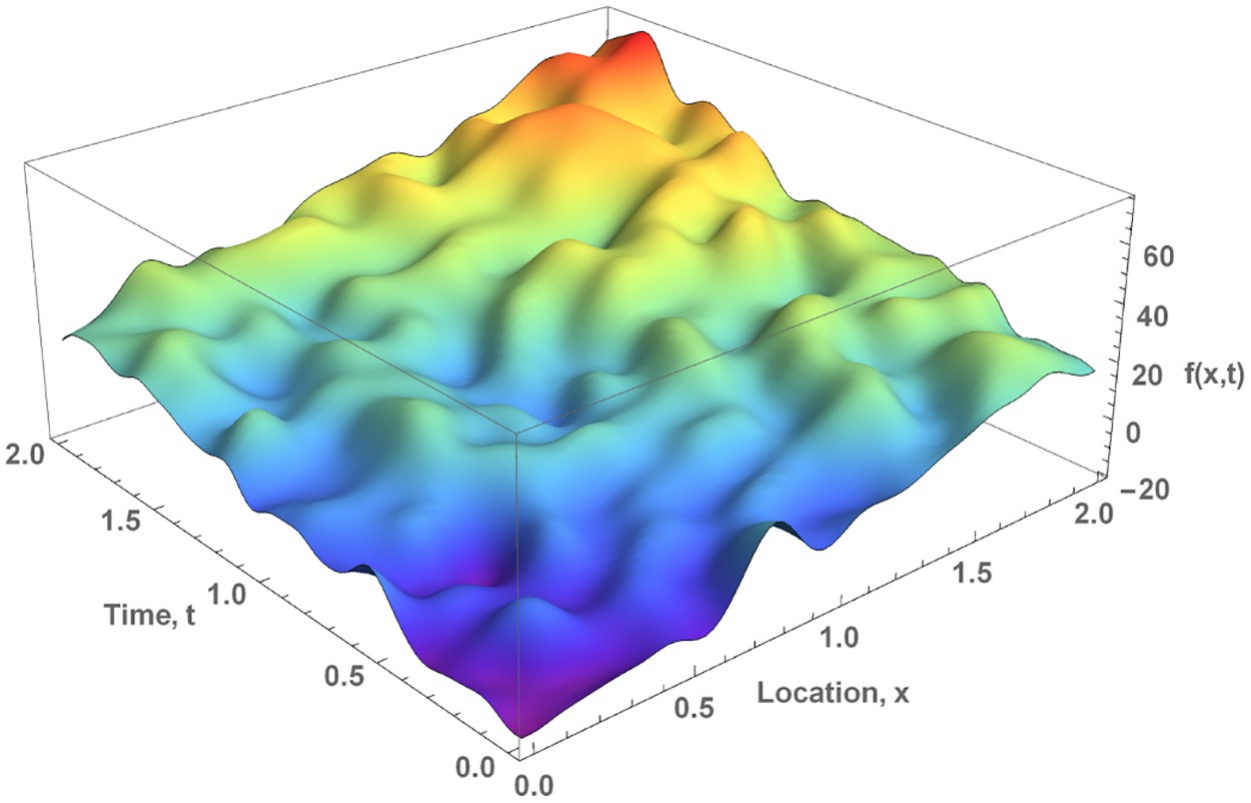
Realisation of a Gaussian random field generated by the squared exponential kernel.

A random field is a generalization of a stochastic process, in which the underlying parameter is a multidimensional vector. Fig 6 shows a realisation of a Gaussian random field generated by the squared exponential kernel, which can be seen as a representation of the evolution of a component the wind velocity in space and time.

A GPR model addresses the question of predicting the value of a scalar variable *y* given the predictor variable **x**. Given a linear regression model of the form

$$\begin{array}{c} y=x^{t}\beta +\varepsilon ,\;\varepsilon ∼N\left(0,\sigma^{2}\right), \end{array}$$ (1)

where the parameters ***β*** and the error variance *σ*^2^ are estimated from the data, the GPR model predicts the variable *y* by introducing two new features:

- A latent random variable *f*(**x**) from a Gaussian process. For any collection of points **x**_1_, **x**_2_, …, **x**_*n*_, it is assumed that *f*(**x**_1_), *f*(**x**_2_), *f*(**x**_3_), …, *f*(**x**_*n*_) are jointly Gaussian distributed random variables with zero-mean and covariance function *k*(**x**, **x**′).
- A basis function *h*, which projects the inputs **x** into a *p*–dimensional feature space.

Combining these features, the GPR model can be formulated as:

$$\begin{array}{c} y=h{\left(x\right)}_{1\times p}^{t}\beta_{p\times 1}+f\left(x\right)+\varepsilon . \end{array}$$ (2)

Under these assumptions, it can be shown that the predicted variable *y* at a point **x**, given a training data set that relates *y* and **x**, is also Gaussian distributed [28]. This characteristic makes GPR a powerful tool, since it is able to give not only an estimation but also its probability distribution. This enables, for example, the provision of a confidence interval for the estimates.

### 3.1 Multiple output Gaussian process regression

The generalisation of the GPR to Multi-Output Gaussian Process Regression (MOGPR) is not straightforward and still a field of active research [29]. The idea behind the MOGPR is to use the knowledge of the relation among the outputs, if any. Typically, this is carried out by adding to the model a formulation of the covariance function that describes not only the auto-correlation of a response variable, but also the correlation among the response variables [30, 31]. However, most GPR implementations usually model only a single response variable due to the difficulty of formulating a covariance function for multiple correlated response variables [32]. An incorrect structure of the covariance matrix can significantly reduce the efficiency of both uncertainty quantification and forecast [33]. In addition, it is difficult to describe the MOGPR process in order to correctly structure the outputs and, at the same time, ensure positive definiteness of the resulting covariance matrices [28]. Therefore, in practice, a single response variable has been considered in many applications (see e.g. [34, 35]). Moreover, popular numerical computation software, such as MATLAB, only provide the single response GPR implementation, whereas the MOGPR is modelled considering the response variables as independent variables, i.e., without taking into account their correlation. The same happens in the case of the Python library scikit-learn. In brief, there is not a single and straightforward generalisation of the GPR to multi-output processes. The approach followed in this paper to solve this problem is described in the next section.

### 3.2 Adaptation of Gaussian process regression to the wind velocity multi-output

As mentioned earlier, the problem studied in this paper is to predict the *u* and *v* components of the wind velocity using the GPR method, which makes the problem a multi-output problem. To extend the single output GPR method to the case with multiple outputs, the following four approaches have been tested:

1. The *u* and *v* components are straightforwardly considered as the outputs: (*y*_1_, *y*_2_) = (*u*, *v*).
2. The *u* and *v* components are calculated in two steps. First, the component *u* is predicted. Then, the component *v* is predicted using the component *u* as a predictor variable: (**x**^*t*^, *u*) → *v*.
3. The outputs are defined as (*y*_1_, *y*_2_) = (*r*, cos (*α*/2)), where *r* is the wind speed and *α* the direction of the wind vector. Then, the *u* and *v* components are restored by computing *α* = 2 arccos(*y*_2_) and (*u*, *v*) = (*r* cos *α*, *r* sin *α*).
4. Three outputs are considered, namely (*y*_1_, *y*_2_, *y*_3_) = (*r*, cos *α*, sin *α*). Then, the *u* and *v* components are restored by computing: (*u*, *v*) = (*r* cos *α*, *r* sin *α*).

The first two approaches directly deal with the estimation of the wind components, whereas the last two approaches split the estimation of the wind speed and direction in two different GPR models. The motivation behind the approaches 3 and 4 is twofold: the wind speed estimation using the GPR method has been proven to be effective in a previous article [19] and the separation of the predictions of wind speed and wind direction may benefit the training of the GPR model, since they are two different physical magnitudes. In the numerical experiments, on average, approach 4 gave the best results in terms of the RMSE, followed by approaches 1 and 3. Approach 2 gave significantly worse outcomes. Notice that, although in the approach 4 one more GPR model must be trained, this does not affect the training time, since this additional training can be done in parallel. Thus, in this paper, the approach 4 has been employed in the numerical experiments reported in Section 4.

## 4 Wind velocity field estimation

In this section, the GPR method proposed in this article for the estimation of the wind velocity field in a given air space will be described. Two different types of estimation are conducted: wind velocity field reconstruction and wind velocity field short-term prediction. The considered air space is the TMA of the LEMD airport. The main features of the proposed method are the following:

- It does not rely on the physics of the atmosphere.
- It does not rely on a discretization of the space, i.e., the wind velocity can be estimated at any point.
- It allows the spatio-temporal relations among observations to be included in the model.
- It allows confidence intervals for the estimates to be computed.
- It requires less than 5 min of computational time on a standard desktop computer.

### 4.1 Implementation and model set up

In this section, the selection of the parameters of the GPR model described in Section 3 will be discussed, which include the error variance *σ*^2^, the basis function and the coefficients *h*(**x**)^*t*^***β***, and the kernel function *k*(**x**, **x**′). The error variance corresponds to the instrumental error, which is assumed to be 9*m*^2^/*s*^2^, according to the typical wind measurement error reported in [18]. The selected basis function

$$\begin{array}{c} h\left(x_{i}\right)={\left(1\;\;x_{i}^{t}\right)}_{5\times 1}^{t} \end{array}$$ (3)

is linear. Thus, the mean of the process changes linearly with respect to the input variables. If a parametric kernel function *k*(**x**, **x**′) is chosen to model the covariance structure, the corresponding hyperparameters must also be estimated. In this paper, the squared exponential kernel [28] is chosen, namely

$$\begin{array}{c} k\left(x_{i},x_{j}|\theta \right)=\sigma_{f}^{2}e^{-r^{2}}, \end{array}$$ (4)

where

$$\begin{array}{c} r=\sqrt{\sum_{m=1}^{d=4}\frac{{\left(x_{im}-x_{jm}\right)}^{2}}{\sigma_{m}^{2}}}, \end{array}$$ (5)

with ***θ*** = (*σ*_*f*_, *σ*_1_, *σ*_2_, *σ*_3_, *σ*_4_) being the hyperparameter vector.

The kernel function (4) is characterised by producing continuous and smooth results. Thus, the GPR model provides a smooth regression. Furthermore, the kernel (4) is universal, i.e., the corresponding GPR method is able to approximate an arbitrary continuous target function uniformly on any compact subset of the input space [36, 37].

With this kernel selection, the correlation between two input variables decreases quickly with the Euclidean distance in space and time. Before computing the distance, each input variable *x*_*im*_ is scaled by a factor $\sigma_{m}^{2}$, to take into account the different scales of the input variables. These scaling factors define how far apart the input data must be so that the response values can be considered uncorrelated. This is an important aspect, since anisotropic variables are present in wind velocity field estimation, i.e., variables that have different length scales [17]. The signal standard deviation *σ*_*f*_ is a factor introduced to adapt the auto-covariance to the output scale. Finally, the coefficients ***β*** and the hyperparameters ***θ*** are estimated in the training phase by the Subset of Data (SD) method [28, Chapter 8]. For a fast and accurate prediction, the Block Coordinate Descent (BCD) approximation is used [38].

### 4.2 Wind velocity field reconstruction

In this section, the ability of the GPR method to reconstruct the wind velocity field within a given air space using historical data is studied. More specifically, the wind velocity field around the LEMD airport is reconstructed for the two wind scenarios described in Section 2.2. A cuboidal region of base size 500 × 500 km centred at the LEMD airport is considered for the reconstruction of the wind velocity field. The altitude ranges from 0.6 km to 14 km.

As said earlier, the data set is split in training and test sets in two different manners, namely randomly selecting a set of individual observations and randomly selecting a set of flights and using all the observations collected during these flights. They will be referred to as randomly splitting the data set by observation and by flight, respectively. To assess the accuracy of the reconstruction, 20% of the available data is reserved for testing as follows:

1. By randomly selecting 20% of the observations. In this case, the wind velocity field reconstruction is conducted over the whole air space.
2. By randomly selecting 20% of the flights. In this case, the wind velocity field reconstruction is carried out along specific aircraft routes.

**Fig 7:**
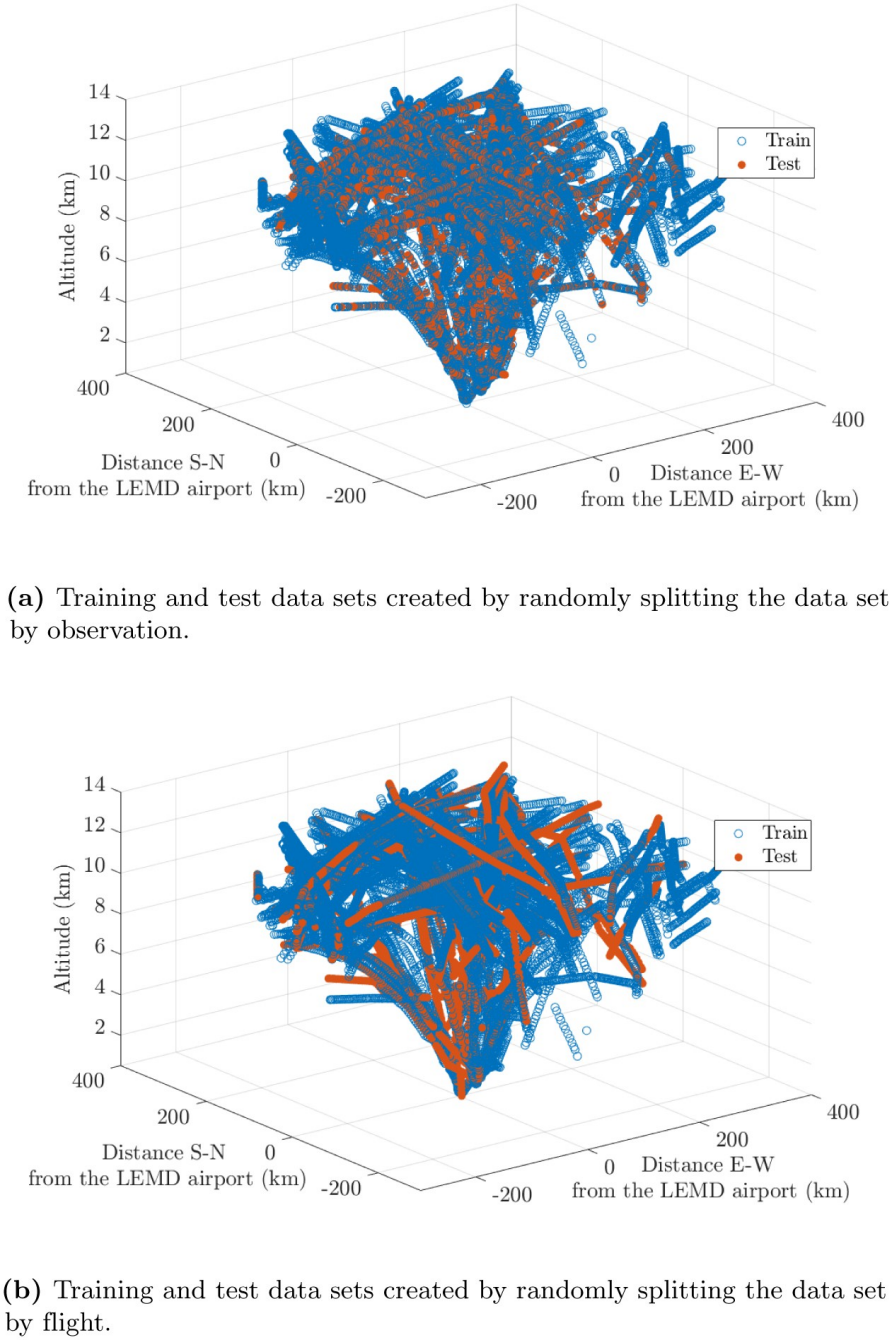
Day 1: Training and test data sets created using observation collected in the period of 1 hour. (**a**) Training and test data sets created by randomly splitting the data set by observation. (**b**) Training and test data sets created by randomly splitting the data set by flight.

The resulting training and test data sets are shown in Fig 7. A model is trained for both the Day 1 and Day 2 data sets using data collected in the period of 1 hour. Both ways for creating the training and test data sets have been considered. The computational time was 5 min on a standard desktop computer.

The estimation errors of the *u* and *v* components of the wind velocity for the Day 1 and the Day 2 data sets are reported in Table 2.

**Table 2:** Wind velocity field reconstruction: Estimation errors for the *u* and *v* components of the wind velocity.

		Data set split by observation		Data set split by flight	
Measure of error	Component	Day 1	Day 2	Day 1	Day 2
RMSE (m/s)	*u*	2.75	1.91	5.88	5.85
	*v*	2.59	1.88	5.58	4.89
MAE (m/s)	*u*	1.49	1.27	4.42	4.27
	*v*	1.46	1.31	3.97	3.71

**Fig 8:**
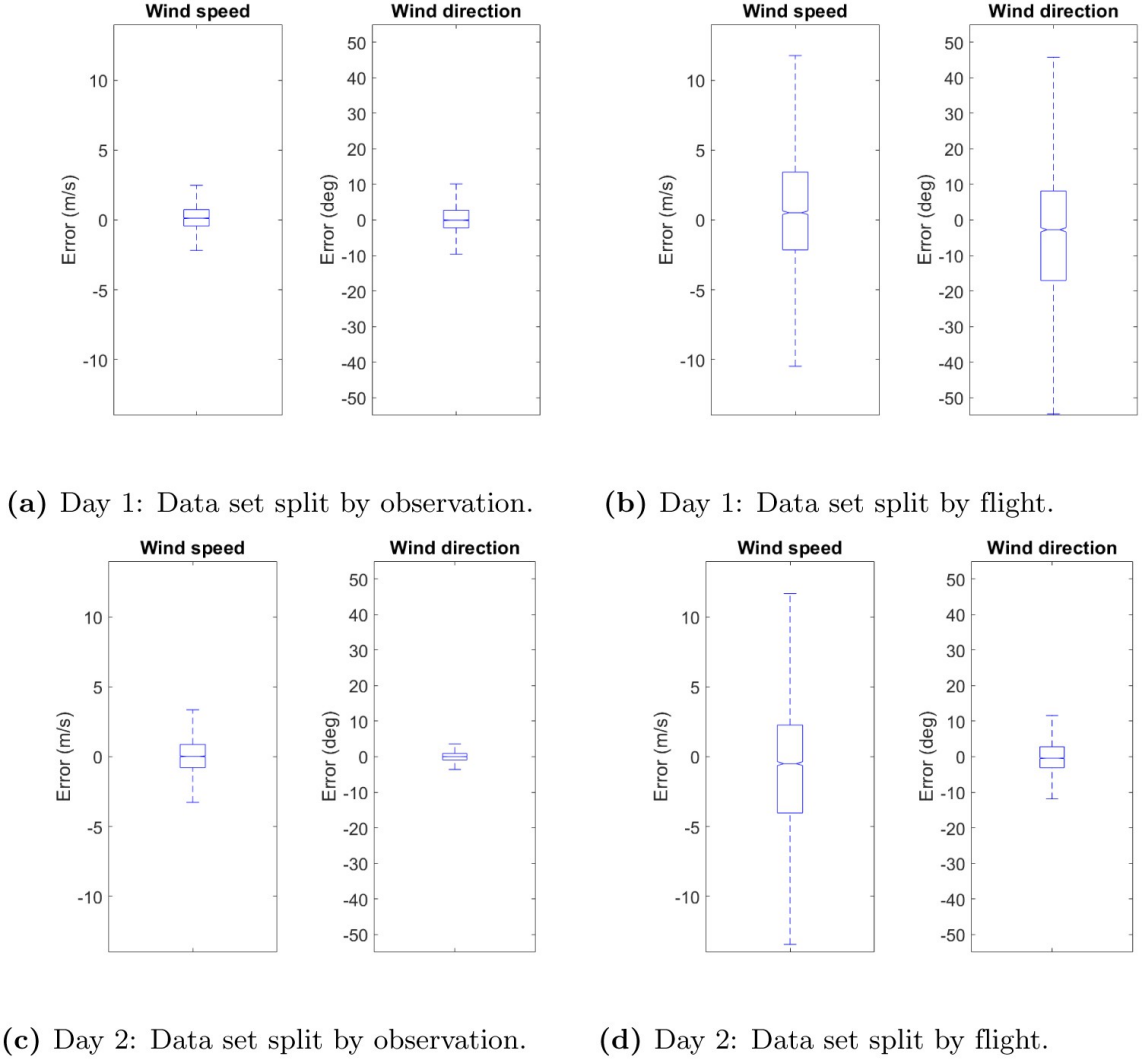
Wind velocity field reconstruction: Boxplots of the estimation errors for the wind speed and wind direction. (**a**) Day 1: Data set split by observation. (**b**) Day 1: Data set split by flight. (**c**) Day 2: Data set split by observation. (**d**) Day 2: Data set split by flight.

The boxplots of the estimation errors for the wind speed and direction are reported in Fig 8. It can be seen that, in general, the estimation errors are unbiased and symmetric. Moreover, there is no remarkable difference between the estimation errors of the *u* and *v* components of the wind velocity obtained for the Day 1 and Day 2 data sets. A similar behaviour is observed for the wind speed. Nevertheless, as expected, the estimation errors of the wind direction are higher for the Day 1 data set, which is characterised by low wind speeds. This is due to the fact that the significance of the wind direction is little when the wind speed is low and negligible when the wind speed is close to zero. Taking into account that, on Day 1, the wind speed is less than 10 m/s for 30% of the data, higher estimation errors in the wind direction can be considered acceptable.

Regarding the method employed for creating the training and test data sets, it has noticeable influence on the results. When the data set is randomly split by observation, the observations are closer in time and space, whereas, when the data set is randomly split by flight, the samples are more separated. Therefore, higher estimation errors are obtained when the training and test data sets are created by randomly splitting the data set by flight.

A similar analysis has been carried out in [12], in which only the wind speed errors have been reported, which have been obtained using training and testing data sets created by randomly spitting by observation. More specifically, the analysis has been conducted employing the so-called meteo-particle model over a set of data collected in a period of 30 min. It can be seen in [12, Table 3] that the obtained Mean Absolute Error (MAE) of the wind speed is similar to the one obtained using the GPR method.

**Fig 9:**
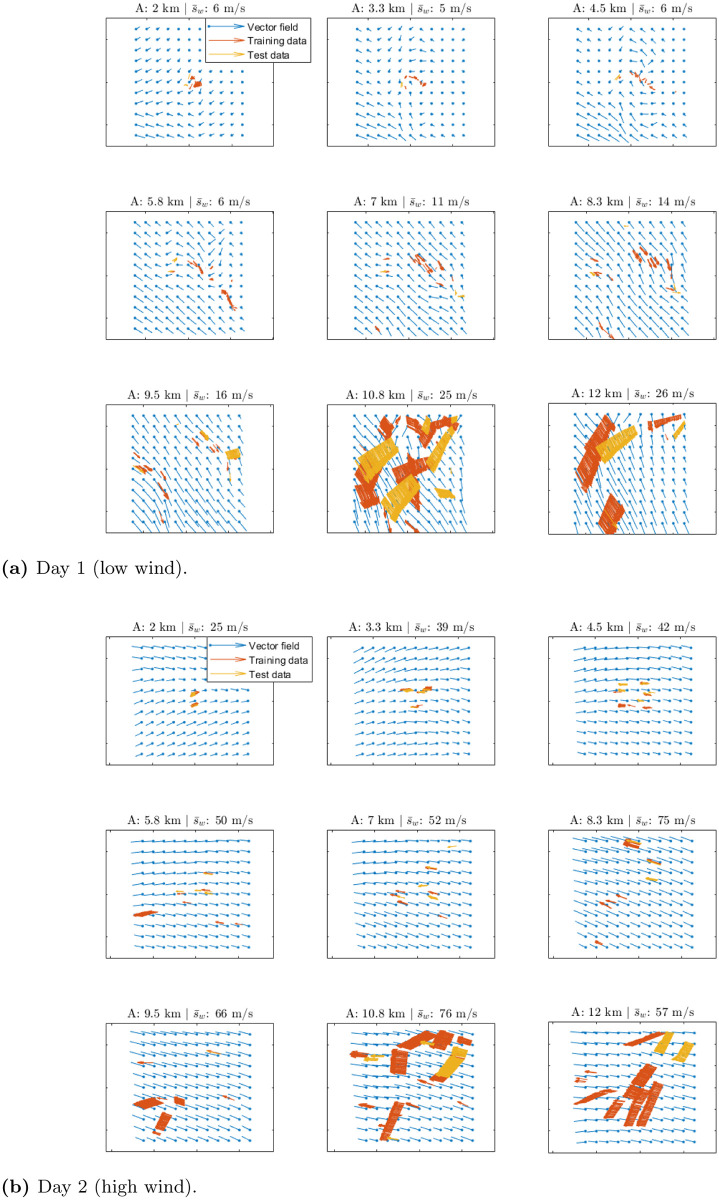
Wind velocity field reconstruction: Reconstructed wind velocity field obtained using the GPR method for different altitudes (A) together with a selection of members of the training and test data sets and the mean wind speed ${\bar{s}}_{w}$. (**a**) Day 1 (low wind). (**b**) Day 2 (high wind).

The reconstructed wind velocity field obtained at a specific time instant using the GPR method is shown in Fig 9 for different altitudes together with a selection of members of the training and test data sets and the mean wind speed ${\bar{s}}_{w}$. It can be seen that the reconstructed vector velocity field is smooth and fits well the data.

### 4.3 Wind velocity field short-term prediction

In this section, the ability of GPR method to perform wind velocity field short-term prediction is studied. More specifically, the wind velocity field around the LEMD airport is predicted in the two wind scenarios described in Section 2.2. In order to perform these short-term predictions, the GPR model is trained every 15 min using data of the previous hour. This process is repeated several times and several measures of error are calculated, which are summarised in Table 3. It can be seen in Table 3 that, as expected, the estimation error is slightly higher than the estimation error obtained in wind velocity field reconstruction using the data set randomly split by flight reported in Table 2. This is due to the fact that the GPR model only has information on past states of the wind, which increases the estimation uncertainty.

**Table 3:** Wind velocity field prediction: Estimation errors for the *u* and *v* components of the wind velocity field.

Measure of error	Component	Day 1	Day 2
RMSE (m/s)	*u*	5.64	7.33
	*v*	5.48	6.31
MAE (m/s)	*u*	4.52	5.93
	*v*	4.46	5.15
MAD (m/s)	*u*	3.11	3.69
	*v*	3.18	3.68

In Table 3, the Median Absolute Deviation (MAD) of the prediction error is also reported. It can be seen that it is lower than both the RMSE and the MAE, because this measure of error is more robust to extreme values in the data set.

Taking into account the presence of a noise of around 3 m/s in the data due to the instrumental error, it can be concluded that the GPR method gives short-term predictions with reasonable estimation errors.

**Fig 10:**
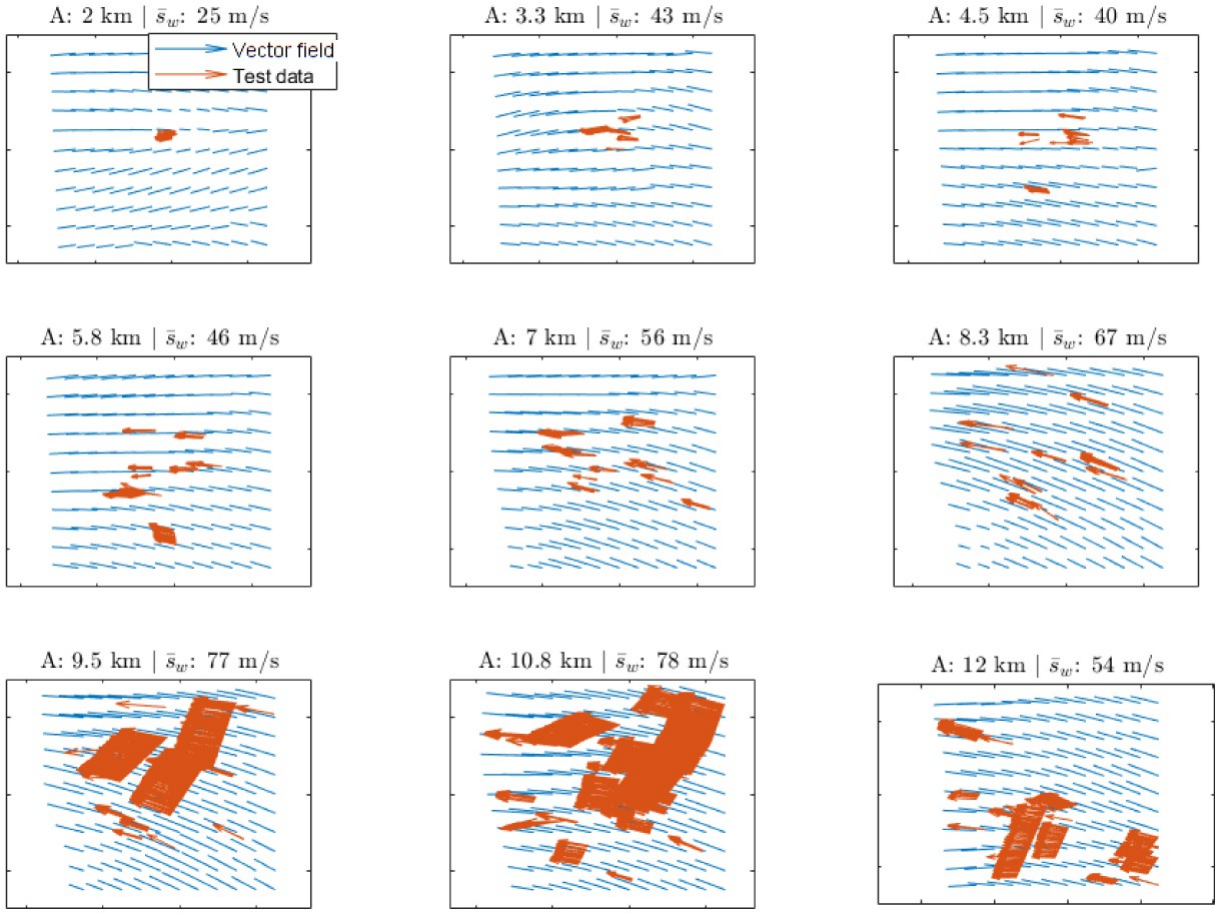
Wind velocity field prediction: A 10 minute ahead wind velocity field prediction for different altitudes (A) using the Day 2 data set (high wind), together with a selection of members of the test data set and the mean wind speed ${\bar{s}}_{w}$.

**Fig 11:**
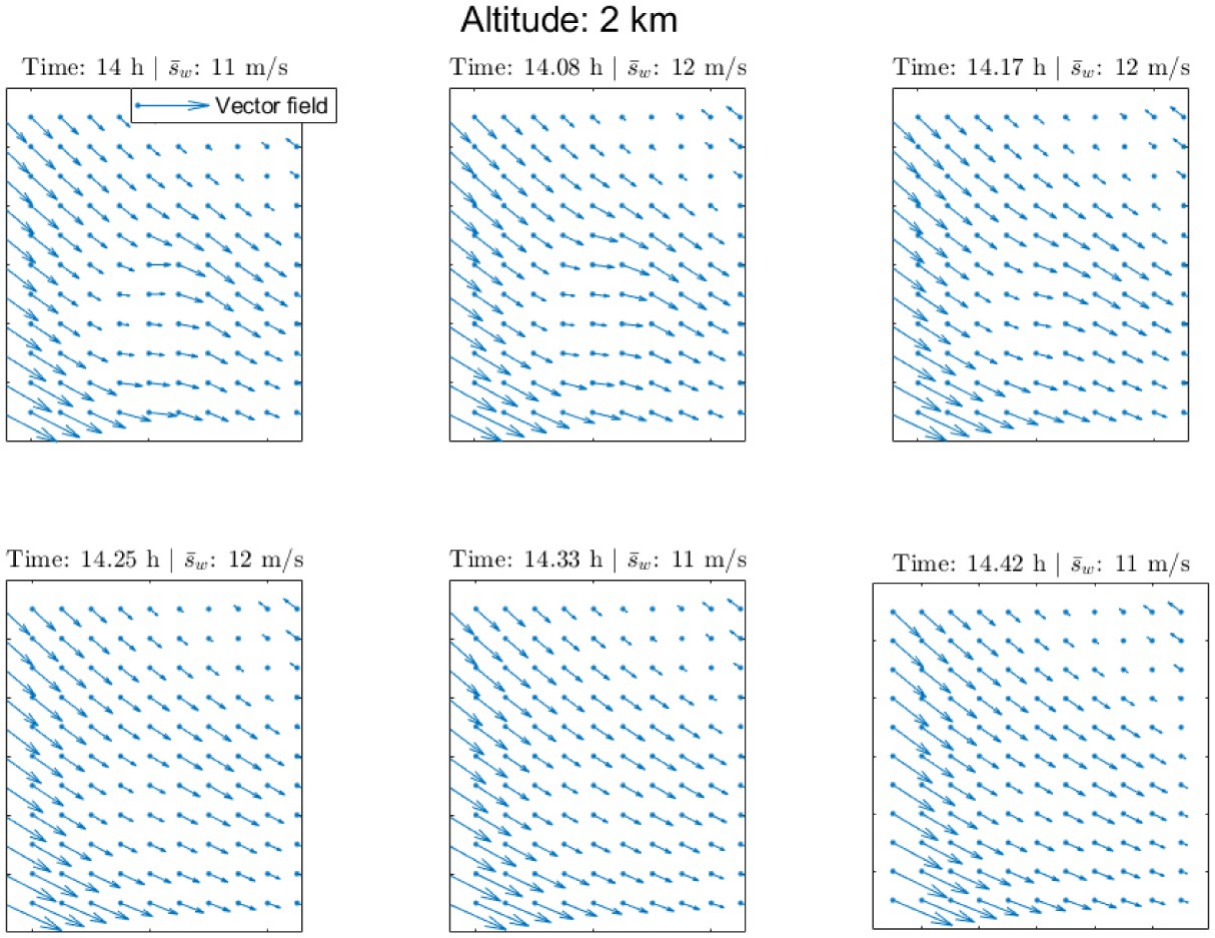
Wind velocity field prediction: Wind velocity field predictions with different time horizons using the Day 1 data set (low wind) together with the mean wind speed ${\bar{s}}_{w}$.

A 15 minute ahead wind velocity field prediction using the Day 2 data set is shown in Fig 10 for different altitudes, together with a selection of members of the test data set, whereas the evolution of a series of wind velocity field predictions with different time horizons using the Day 1 data set is shown in Fig 11. The prediction evolves over time and stabilises, since the correlation in time, which is governed by the kernel in Eq (4), decreases rapidly.

**Fig 12:**
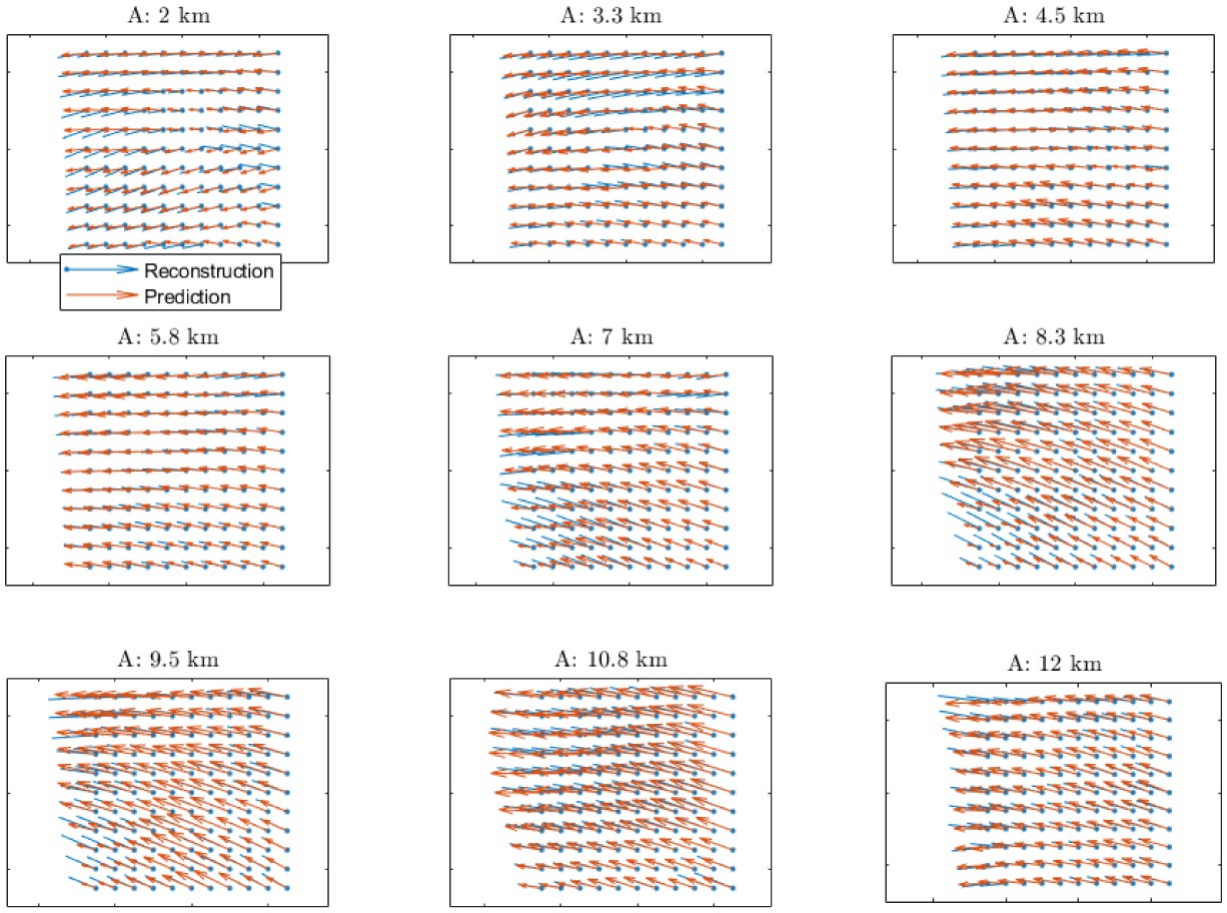
Comparison between the predicted and reconstructed wind velocity fields at different altitudes (A).

Another way to evaluate the precision of the prediction is to compare the predicted wind velocity field with the reconstructed wind velocity field for the same time instant. A comparison of a 20 minute ahead wind velocity field prediction with the corresponding wind velocity field reconstruction is represented in Fig 12 for different altitudes. It can be observed that the wind velocities largely agree between them.

**Fig 13:**
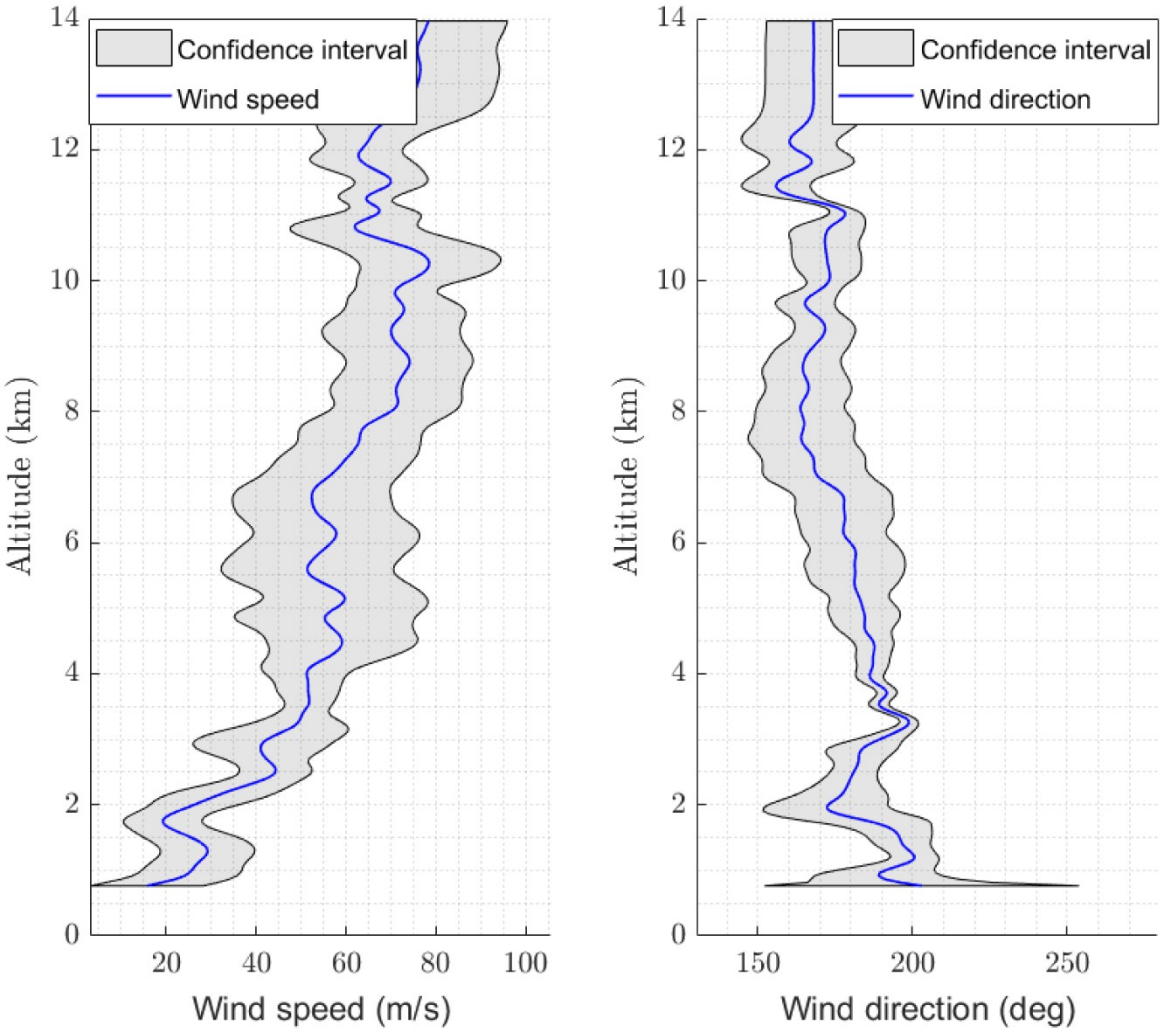
Wind velocity field prediction: 10 minute ahead prediction of the wind speed and wind direction along the vertical at the RILKO IAF point, together with the corresponding 2 standard deviation confidence intervals. This prediction has been calculated using the Day 1 data set.

As mentioned in Section 3, the GPR method allows the confidence intervals for the wind velocity field estimates to be computed. The 10 minute ahead prediction of the wind speed and wind direction along the vertical at a specific point close to the LEMD airport, known as RILKO IAF, together with the corresponding 2 standard deviation confidence intervals are shown in Fig 13. This prediction has been calculated using the Day 1 data set. The percentage of estimates that lie within the GPR confidence intervals has also been computed for both data sets. For the Day 1 data set, the percentage of estimates that lie within the GPR confidence intervals is 88.69%, whereas this percentage is 86.90% for the Day 2 data set. Notice that, if the estimates were normally distributed, approximately 95% of the observations would lie within the 2 standard deviation confidence intervals. However, since in this case the aircraft derived wind velocity field does not behave as a Gaussian field, the GPR model is overconfident. Thus, the width of the confidence interval should be increased if 95% is to be achieved.

In conclusion, the GPR method is able to reconstruct the wind velocity field and to provide accurate short-time predictions of the wind velocity field. Furthermore, the GPR method has been generalised to estimate not only the wind speed but also the wind direction without affecting the accuracy of the wind speed estimation. Actually, the magnitude of the wind speed errors is similar to the one reported in [19]. It will be shown in the next section that the results of the wind velocity field estimation also agree with the ECMWF ERA5 reanalysis data.

## 5 Validation of the Gaussian process regression model

In this section, the GPR model is validated by comparing the estimates obtained using this method with the ECMWF ERA5 reanalysis data, which have been considered as the reference for validation. The ECMWF ERA5 reanalysis data base yields global atmospheric reanalysis data for all altitude levels with a 0.25 degrees resolution in both latitude and longitude.

A comparison between the Day 1 and Day 2 aircraft derived data sets with the ECMWF ERA5 reanalysis data is shown in Table 4. For this comparison, the differences have been computed for every hour ranging from 09:00 to 15:00 UTC, with a tolerance of 15 min in time and 1000 ft in altitude. The ECMWF ERA5 data, which are given at grid points, have been interpolated linearly to compute the corresponding values at the locations at which aircraft derived wind observations are available. The differences are small but significant. Despite these differences, it is expected that the estimates obtained with the GPR method and the reanalysis data agree on average. Special attention has been paid to the spatio-temporal locations where no aircraft derived data are available, namely low altitudes and outside the air traffic routes. For this purpose, the comparison has been conducted as follows:

1. The ECMWF ERA5 reanalysis data corresponding to February 23, 2020 (Day 1) and December 21, 2019 (Day 2) for the relevant air space have been read-off from the data base.
2. For each day, different time instants and altitudes have been considered. More specifically, the ECMWF ERA5 reanalysis data corresponding to 09:00, 12:00, and 15:00 UTC for altitudes 5.6, 9.3, 10.5, 11.2, 12, and 12.9 km have been considered.
3. The relevant airspace is modelled as a cuboidal region of base size 500 × 500 km centred at the LEMD airport. The GPR model has been trained for every hour using aircraft derived data observed in this airspace.
4. After the training phase, the GPR method was used to perform an estimation at each grid point of the ECMWF ERA5 reanalysis data set. Then, the estimates have been compared with the ECMWF ERA5 reanalysis data and error measures have been computed.

**Table 4:** Validation of the GPR model: Comparison between the aircraft derived data and the ECMWF ERA5 reanalysis data.

Measure	Variable	Day 1	Day 2
Bias (m/s)	Wind speed	2.4	-0.51
MAE (m/s)	Wind speed	5.91	5.72
Bias (deg)	Wind direction	3.87	2.24
Variance (%)	Wind direction	15.16	0.65

**Fig 14:**
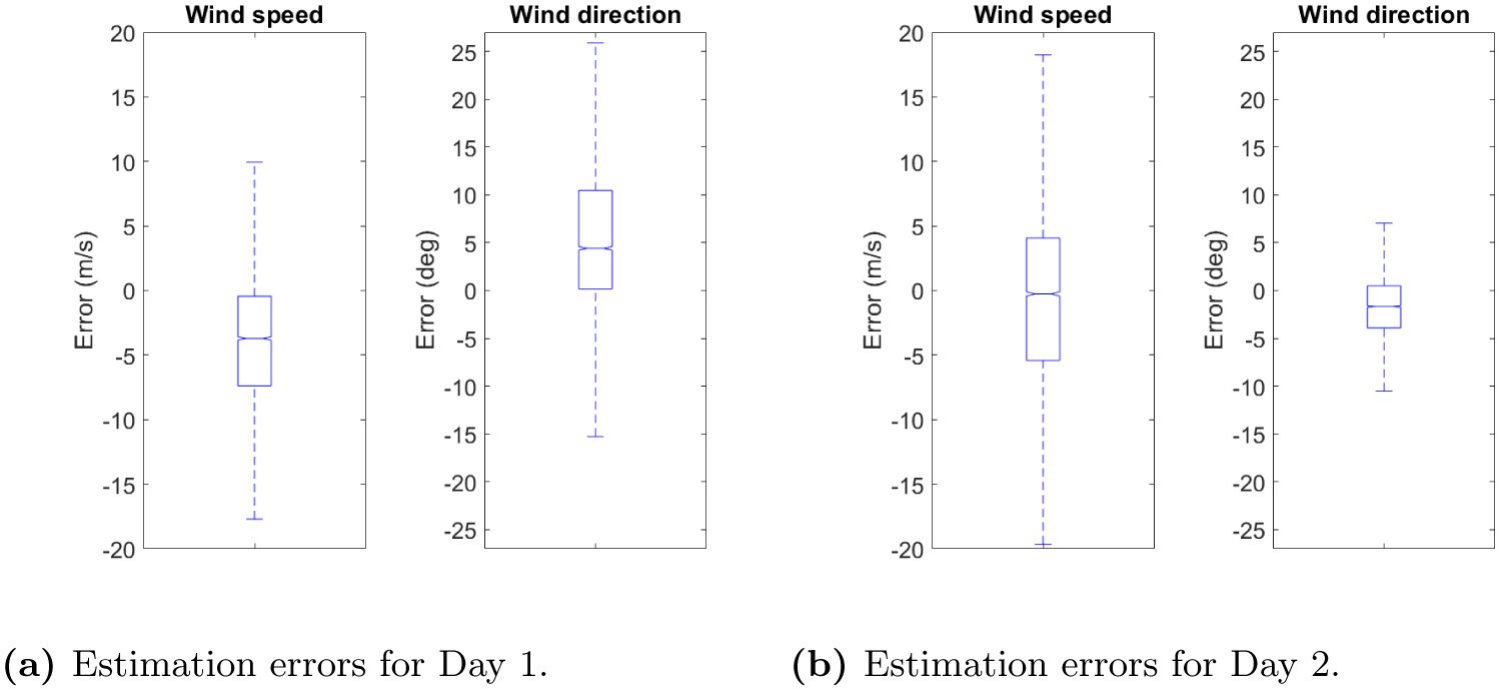
Validation of the GPR model: Boxplots of the estimation errors for the wind speed and direction obtained with the GPR method with respect to the ECMWF ERA5 reanalysis data. (a) Estimation errors for Day 1. (b) Estimation errors for Day 2.

Fig 14 shows the boxplots of the differences between the estimates of the wind speed and direction obtained with the GPR method and ECMWF ERA5 reanalysis data for Day 1 and Day 2. It can be seen that, for both days, the errors are small. Furthermore, as shown in Table 5, these errors are smaller than those between the aircraft derived data and the ECMWF ERA5 reanalysis data reported in Table 4. This is due to the fact that the GPR method yields wind velocity field estimates that are smoother than the aircraft derived data. Moreover, the GPR model acts as a noise filter, since measurement noise is included in the model represented by Eq (2).

**Table 5:** Validation of the GPR model: Comparison between the estimates obtained with the GPR method and the ECMWF ERA5 reanalysis data.

Measure	Variable	Day 1	Day 2
Bias (m/s)	Wind speed	-3.84	-1.01
MAE (m/s)	Wind speed	5.10	6.06
Bias (deg)	Wind direction	4.33	-1.56
Variance (%)	Wind direction	4.96	0.28

As mentioned in Section 2.2, most of the aircraft derived data are located at cruise altitude. Moreover, low altitude data are in general located close to the LEMD airport, as shown in Figs 2 and 7. The interquartile ranges of the estimation errors, on Day 1, at different altitudes and horizontal distances to the LEMD airport, are shown in Table 6. The interquartile ranges of the estimation errors at different horizontal distances to the LEMD airport have been computed for the altitude of 9.4 km. It can be seen that, at cruise altitude (ranging from 10.6 km to 11.2 km), the discrepancy between the GPR estimates and the ECMWF ERA5 reanalysis data is lower than at other altitudes, except for the wind speed estimates at altitude 5.4 km, which is due to the significant low speed observed at this altitude on Day 1. Moreover, excluding the area surrounding the LEMD airport, in which the number of observations is significantly higher, similar discrepancies between the GPR estimates and the ECMWF ERA5 reanalysis data are observed at different horizontal distances.

**Table 6:** Validation of the GPR model: Interquartile ranges of the estimation errors at different altitudes and horizontal distances to the LEMD airport for Day 1.

	Altitude (km)						Horizontal distance to the LEMD airport (km)					
Variable	5.8	9.4	10.6	11.2	12	12.9	60	120	180	240	300	360
Wind speed (m/s)	4.7	6.1	6.2	5.3	7	6.4	4.5	5.4	5.8	6.15	7.5	7.1
Wind direction (deg)	34	12.4	7.7	7.3	12.2	15.1	7.6	10	11.3	12.4	10.5	8.7

Thus, it is possible to conclude that the estimates obtained using the GPR method agree with the ECMWF ERA5 reanalysis data even when there is no aircraft derived wind observation near the ECMWF ERA5 reanalysis grid point, which shows the capability of the GPR method to perform wind velocity field estimation at locations in the surroundings of which no aircraft derived wind observation is available.

In order to test the effect of outliers removal from the raw data set, the GPR models have been trained using both the raw data and the processed data, and their wind velocity field estimates have been compared to the ECMWF ERA5 reanalysis data. The results of the comparison are shown in Table 7. It can be seen that, for the Day 1 data set, similar statistics for the wind speed and direction are obtained using both the raw data and the processed data. On the contrary, for the Day 2 data set, similar statistics are obtained only for the wind direction whereas, significant differences are present in the statistics for the wind speed. More specifically, the Bias and the MAE increase considerably when the outliers are not removed from the raw data set.

**Table 7:** Validation of the GPR model: Comparison between the estimates obtained with the GPR method, with and without outliers, and the ECMWF ERA5 reanalysis data.

		Processed data		Row data	
Type	Variable	Day 1	Day 2	Day 1	Day 2
Bias (m/s)	Wind speed	-3.84	-1.01	-3.87	-4.01
MAE (m/s)	Wind speed	5.10	6.06	4.87	10.31
Bias (deg)	Wind direction	4.33	-1.56	5.2	-1.31
Variance (%)	Wind direction	4.96	0.28	5.27	0.80

## 6 Discussion

In this paper, a method based on GPR for wind velocity field reconstruction and short-term prediction has been presented. The results of the numerical experiments show that the proposed GPR method is effective in modelling the spatio-temporal wind correlation. The GPR method proposed in this paper is fast, allowing for data assimilation. Unlike the grid-based methods, in which the dimensions of the grid require large matrix operations, the GPR method straightforwardly yields estimates at any location. The proposed method is accurate. It has been tested in different scenarios, achieving similar performances and showing its capability to precisely estimate the wind velocity field at different spatial locations and at different time instants. The GPR model has been validated by comparing the estimates obtained using this method with the ECMWF ERA5 reanalysis data. The results of the validation show that they are consistent with the ECMWF ERA5 data even in those regions in which the data coverage is low or inexistent, i.e., the obtained wind velocity field estimates largely agree with unobserved data. The proposed method for wind velocity field estimation, which can rapidly assimilate new observations to provide precise wind estimates at any location and time, improves aircraft trajectory predictability, which is a key aspect for TBO, the central element of the future ATM paradigm.
